# An Orthologous Epigenetic Gene Expression Signature Derived from Differentiating Embryonic Stem Cells Identifies Regulators of Cardiogenesis

**DOI:** 10.1371/journal.pone.0141066

**Published:** 2015-10-20

**Authors:** Brian W. Busser, Yongshun Lin, Yanqin Yang, Jun Zhu, Guokai Chen, Alan M. Michelson

**Affiliations:** 1 Systems Biology Center, Division of Intramural Research, National Heart Lung and Blood Institute, National Institutes of Health, Bethesda, MD, 20892, United States of America; 2 Center for Molecular Medicine, National Heart Lung and Blood Institute, National Institutes of Health, Bethesda, MD, 20892, United States of America; Laboratoire de Biologie du Développement de Villefranche-sur-Mer, FRANCE

## Abstract

Here we used predictive gene expression signatures within a multi-species framework to identify the genes that underlie cardiac cell fate decisions in differentiating embryonic stem cells. We show that the overlapping orthologous mouse and human genes are the most accurate candidate cardiogenic genes as these genes identified the most conserved developmental pathways that characterize the cardiac lineage. An RNAi-based screen of the candidate genes in *Drosophila* uncovered numerous novel cardiogenic genes. shRNA knockdown combined with transcriptome profiling of the newly-identified transcription factors zinc finger protein 503 and zinc finger E-box binding homeobox 2 and the well-known cardiac regulatory factor NK2 homeobox 5 revealed that zinc finger E-box binding homeobox 2 activates terminal differentiation genes required for cardiomyocyte structure and function whereas zinc finger protein 503 and NK2 homeobox 5 are required for specification of the cardiac lineage. We further demonstrated that an essential role of NK2 homeobox 5 and zinc finger protein 503 in specification of the cardiac lineage is the repression of gene expression programs characteristic of alternative cell fates. Collectively, these results show that orthologous gene expression signatures can be used to identify conserved cardiogenic pathways.

## Introduction

The post-translational covalent modifications of the histone proteins that comprise the nucleosome have been shown to be associated with either transcriptional activation or repression [1]. Recently, numerous studies have shown that the distribution of the epigenetic modifications of the histone proteins can be used as predictors of *cis* regulatory element (CRE) activity [2].

Embryonic stem cells (ESCs) can be differentiated into numerous distinct cell types *in vitro* including cardiovascular cells [3]. This finding has provided applications in regenerative medicine and serves as an experimental system for studying human developmental mechanisms. In fact, the directed differentiation of ESCs along the cardiac lineage recapitulates aspects of embryonic development with the stereotyped appearance of precursor and differentiated cell populations having distinctive markers. Further, these cell populations are easily accessed for epigenomic and transcriptomic analyses and genetic manipulation. Recent studies have taken advantage of these attributes to characterize the histone mark distribution and expression profiles of differentiating human and mouse ESCs to uncover the CREs and transcript patterns that characterize the mammalian cardiac lineage [4,5].

Here we used a predictive multi-species epigenetic signature of differentiating mouse and human ESCs along the cardiac lineage in combination with an RNAi-based screen in *Drosophila* and shRNA knockdown and transcriptome profiling to identify and characterize novel cardiogenic genes. We show previously uncharacterized roles for the transcription factors (TFs) zinc finger protein 503 (ZNF503), zinc finger E-box binding homeobox 2 (ZEB2) and NK2 homeobox 5 (NKX2-5) in the specification and differentiation of the mammalian cardiac lineage.

## Materials and Methods

### Analysis of ChIP experiments

The coordinates of genomic regions defined as enriched for a particular histone modification were identified using MACS by comparing to input sequence with default parameters, and were required to be identified in the replicate ChIP-seq experiments (with at least 100 bp overlap) [6]. Genomic regions were considered enriched for multiple histone modification if at least 100 bp of these sequences overlapped. For human candidate genes, regions marked by H3K27me3 at the ESC state followed by H3K4me3 and H3K36me3 as either a tripotential cardiovascular progenitor or a committed cardiovascular cell were identified and for mouse candidate genes, regions marked by H3K4me1 and H3K27me3 at the ESC state and H3K4me1 and H3K27ac as a CP were identified. These CREs were annotated to genes using GREAT with standard parameters [7]. CREs were associated with its appropriate target gene if one of its neighbors showed increased expression of at least 2-fold from the ESC state to the cardiac precursor state using previously analyzed expression profiles [4,5]. Over-represented GO categories were identified with FuncAssociate2.0 and standard parameters [8]. Orthologous gene predictions were performed using DIOPT [9]. Pathway enrichment analysis was performed using Reactome [10].

### Maintenance of Human Embryonic Stem Cells and Cardiovascular Directed Differentiation

H1 embryonic stem cells (WA01, US National Institute of Health (NIH), human ESC registry no. 0043) were grown on matrigel-coated plates (10 μg/cm^2^) in E8 media (Essential 8 Medium, Life Technologies) which was changed daily and passaged with 0.5 mM EDTA in PBS plus 0.45% NaCl according to published procedures [11,12]. Differentiation of H1 ESCS along the cardiac lineage was performed in E8 basal medium in a protocol modified from a previous study [13]. Briefly, H1 ESCs were grown to ~80% confluence in E8 media. The entire time course of differentiation was performed in differentiation basal medium (E8 medium (minus FGF2, TGFβ and insulin), 1X Chemically Defined Lipid Concentrate (Life Technologies) and 1X Pen-Strep (Life Technologies)). Cardiac lineage differentiation was induced first with the addition of GSK3β inhibitor CHIR99021 (5 μM, Tocris) for the first 24 hours followed by days 2 through 5 the Wnt inhibitor IWP2 (3 μM, Tocris). Insulin (20 μg/ml, Sigma) was added at day 7 to maintain cardiomyocytes (CMs).

### shRNA knockdown

The production of lentivirus for shRNA transformation of human ESCs followed standard procedures (http://dharmacon.gelifesciences.com/shrna/gipz-lentiviral-shrna/). Virus was produced in 293T cells and used to infect H1 ESCs (O/N), cells were allowed to recover followed by selection with puromycin (1 μg/ml, O/N). Five shRNA knockdown vectors for each gene were tested using GIPZ Lentiviral shRNA (GE Healthcare). The shRNA line with the best knockdown efficiency as determined by real-time PCR was used for all future differentiations. Cells infected with lentiviral shRNAs targeting GFP or empty vector were used for WT human ESCs.

### Flow Cytometry

Cells were singularized using TrypLE (Invitrogen), fixed in 1% paraformaldehyde (20 min.) and stained in PBS plus 0.5% BSA and 0.1% Triton-X-100 [14]. cTnT antibody (Lab Vision, ms-295-p1) was used at 1:200 and detected with Ax488 Goat anti-Mouse IgG (Life Technologies) diluted to 1:1000. Viability was assessed with Fixable Viability Dye (eBioscience). Data was collected on a BD LSRFortressa and analyzed with BD FACSDiva Software.

### Transcriptomic analyses

RNA was isolated from cells with Trizol followed by removal of contaminating genomic DNA by TURBO DNA-free kit (Life Technologies). For real-time PCR assays, cDNA was prepared with Maxima RT (Thermo Scientific) and random pentadecamers (Operon). SYBR Green PCR master mix (Applied Biosystems) was used for real-time PCR. For RNA-seq, two samples for both WT and each shRNA knockdown condition were isolated at days 0, 2, and 6, and two (NKX2-5 shRNA) or four (WT and ZEB2 shRNA) samples were isolated at day 10 following directed differentiation along the cardiac lineage. RIN scores varied from 7.5 to 8.3 for all samples except those from day 10 ZNF503 shRNA cells (Agilent 2100 Bioanalyzer). Library preparation was performed with 1 μg of DNA-free RNA using TruSeq Stranded Total RNA Library Prep Kit (Illumina). The resulting libraries were sequenced on an Illumina HiSeq2500 platform. The paired-end raw reads were mapped to the human genome (hg19, GRCh37) by Tophat 2.0.14 (bowtie v2.2.1.0, samtools v0.1.19.0) allowing 2 mismatches and 1 best hit (http://ccb.jhu.edu/software/tophat/index.shtml). We counted the mapped reads in individual refSeq gene region of each sample using RSeQC v2.6.1 (http://rseqc.sourceforge.net/). Read coverage for all samples (excluding the discarded ZNF503 shRNA day 10 samples) averaged 48.7 +/- 5.7 for exons, 37.1 +/- 4.7 for introns, 12.8 +/- 2.4 for intergenic sequences and 1.3 +/- 0.11 for partial sequences. The R Bioconductor edgeR package was used to normalize raw reads and compare the difference between WT and shRNA knockdown conditions at a single time point as well as WT at day 0 compared to day 2, 6 or 10. The differentially expressed genes were selected by fold change and false discovery rate (FDR): day 0 (2-fold changes with 10% FDR), day 2 (4-fold changes with 10% FDR), day 6 (8-fold changes with 1% FDR) and day 10 (8-fold changes with 1% FDR). All differentially-expressed genes across all conditions were clustered using k-means clustering and visualized using Java TreeView (http://jtreeview.sourceforge.net/). All samples (either 2 or 4 replicates) were used to define differentially-expressed genes while only 2 replicates were used in clustering for clarity of presentation. The raw reads from the RNA-seq data were deposited to NCBI GEO database (http://www.ncbi.nlm.nih.gov/geo) under accession # GSE69618. The following real-time PCR primers were used: POU5F1 (GAGAAGGATGTGGTCCGAGT, GTGCATAGTCGCTGCTTGAT), NANOG (CCACTCCTGGAACACTCAGA, CATGCAGGACTGCAGAGATT), MIXL1 (CCCTCTTCCAGGTATGGTTC, GGAAGGATTTCCCACTCTGA), GATA4 (TACCACAAGATGAACGGCAT, GTCTGGCAGTTGGCACAG), NKX2-5 (AGGCGCAGGTCTATGAGC, GTGGACGTGAGTTTCAGCAC), TBX5 (TTCTGCACTCACGTCTTTCC, TGGCAAAGGGATTATTCTCA), MEF2C (GGACAAGGAATGGGAGGATA, CGGTGTTAAACCCAGACAGA), MYL7 (TGAACAAGCCCAGATACAGG, TGGGAGTAGGTCTCCCTCAGO), TNNT2 (ATTCTGGCTGAGAGGAGGAA, CTGCAGGTCGAACTTCTCTG), MYH6 (CCTTTGACATTCGCACTGAG, AACACCTGGTCCTCCTTCAC), MYH7 (CTGCTCTGGAGGCCTTTG, TGTCTGCAGATGCCAACTTT), ZNF503 (TCGCTTTCTGCCCTAAGAAG, CCGGAGCTATTTCCAGAGAG), ZEB2 (TTTCCTGCCCTCTCTGTAGC, ACTTGCGATTACCTGCTCCT) and GAPDH (CGTCTTCACCACCATGGAGA, CGGCCATCACGCCACAGTTT).

### Drosophila

Embryos were collected by crossing UAS-RNAi lines from the TRiP at Harvard Medical School and the VDRC [15,16] to flies expressing TinD-GAL4 and Hand-GAL4 [17,18]. The efficiency of RNAi knockdown was enhanced by both collecting embryos at 29°C and using UAS-dcr2. Embryo collections, fixation and antibody stains followed standard procedures [19–23]. The Zfh1 antibody was used at 1:1500 and was provided by J. Skeath (Washington University, St Louis, MO) and the Tin antibody was created according to previously published procedures and used at 1:500 [18].

## Results and Discussion

### A predictive orthologous chromatin and gene expression signature of differentiating ESCs

Previous studies used the distribution of histone marks and gene expression profiles of differentiating ESCs to identify the CREs and expression patterns that characterize the mouse and human cardiac lineage [4,5]. Here we used these findings to generate a predictive gene expression signature of candidate cardiogenic genes to identify the developmental pathways required for differentiation along the cardiac lineage.

**Fig 1:**
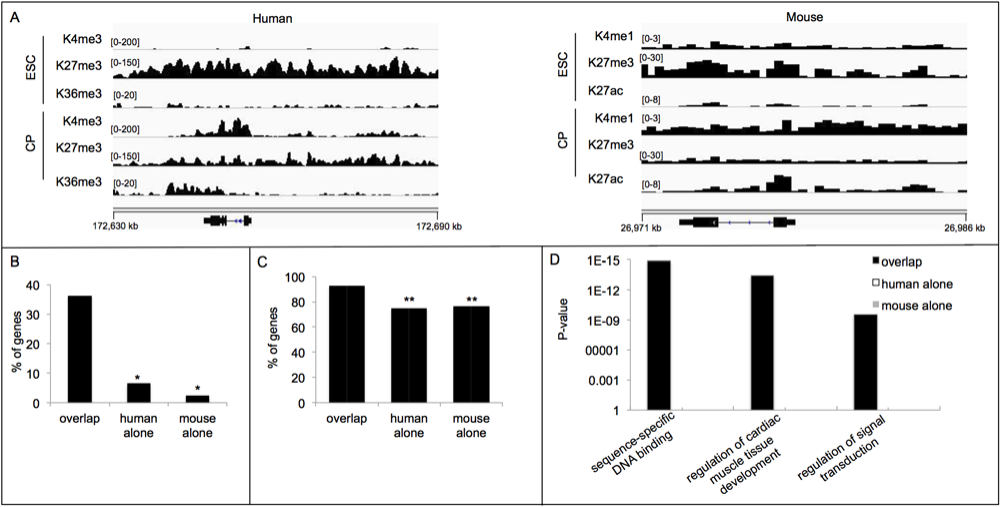
A multi-species epigenetic and gene expression signature identifies candidate cardiogenic genes. (A) The distribution of the indicated histone modifications to the genomic region of the mouse and human NKX2-5 gene at the ESC or CP state. Genomic coordinates are indicated for human chromosome 5 (hg19) and mouse chromosome 17 (mm9). Brackets indicate scale of peak. (B) Percentage of indicated genes known to regulate mammalian cardiogenesis. (C) Percentage of indicated cardiogenic genes conserved in *Drosophila*. (D) Enriched GO categories associated with the indicated genes. * p < 0.0001; **p < 0.0005.

A study by Murry and colleagues used chromatin immunoprecipitation with massively parallel sequencing (ChIP-seq) to identify trimethylation of lysine 4 on histone 3 (H3K4me3), trimethylation of lysine 27 on histone 3 (H3K27me3) and trimethylation of lysine 36 on histone 3 (H3K36me3) in differentiating human ESCs [4]. H3K4me3 is enriched near transcriptional start sites whereas H3K27me3 and H3K36me3 are marks of repressed and active genes, respectively [1]. To identify human cardiogenic genes, we reasoned that such genes will show marks of repression at the ESC state as evidenced by labeling with H3K27me3, and marks of activation when pluripotent cells become restricted as cardiac precursor cells (CPs), as detected by the presence of H3K4me3 and H3K36me3. In addition, expression of these genes should increase as the pluripotent embryonic cells differentiate into CPs (see Materials and Methods). All genes that showed this gene expression signature were identified, with a representative example (the well-known cardiac regulatory factor, NKX2-5) shown in Fig 1A. Additional examples of previously uncharacterized cardiogenic genes are shown in S1 Fig. In total, this approach identified 298 potential human cardiogenic genes. Moreover, GO analysis of these genes revealed enrichment for functions associated with signaling, transcription and cardiac development and specification (S1 Table). Furthermore, in agreement with a preceding study [4], we show that genes identified by a combination of predictive histone mark distributions and appropriate gene expression changes are more often associated with upstream regulatory functions (e.g., signaling and transcription) as well as cell fate specification and cardiac differentiation functions as compared to those identified solely with a predictive histone mark profile (data not shown). This result underscores the utility of combining epigenetic and gene expression data in identifying the regulatory genes critical for orchestrating cell fate decisions. In total, an epigenetic and gene expression signature of differentiating ESCs reliably identified numerous regulators of human cardiac lineage specification.

In a related study, Bruneau and his colleagues used expression profiling and ChIP-seq to identify monomethylation of lysine 4 on histone 3 (H3K4me1), acetylation of lysine 27 on histone 3 (H3K27ac) along with H3K27me3 and H3K4me3 of differentiating mouse ESCs along the cardiac lineage [5]. H3K4me1 is generally associated with enhancer sequences and H3K27ac is associated with active promoters and enhancers. Because of the selective regulation of gene expression by enhancers [24,25], we reasoned that enhancers showing signs of repression in ESCs followed by activation during specification of the cardiac lineage would be an ideal indicator of critical cardiogenic genes. To identify such CREs, we identified all genomic regions that were marked with H3K4me1 and H3K27me3 at the ESC stage and H3K4me1 and H3K27ac at the CP stage. In addition, we associated an enhancer to its regulated target gene by identifying the neighboring gene that increased expression as a CP from the ESC state (see Materials and Methods). In total, this approach identified 477 candidate mouse cardiogenic genes, with a representative example shown in Fig 1A. Similar to the human signature, this approach identified genes enriched for signaling, transcription and cardiac development and specification functions (S1 Table). In total, these studies confirm that an epigenetic and gene expression signature can identify regulators of mouse cardiac lineage specification in differentiating ESCs.

### Predicted cardiogenic genes shared by human and mouse are the most accurate candidates for having cardiac regulatory activity

The preceding epigenetic and gene expression signature identified hundreds of mammalian cardiogenic genes. To identify the functions of these candidate genes, we first identified the orthologous genes shared by human and mouse. This analysis identified 200 genes unique to the human signature, 379 unique to the mouse signature with 98 overlapping candidate genes (S1 Table). We examined if loss-of-function (LOF) mutations in these genes have been previously described to play a role in mouse cardiovascular development [26]. This analysis revealed that the overlapping human and mouse candidate genes were most often associated with cardiovascular phenotypes in the mouse heart (Fig 1B). In addition, as key regulatory genes are more often conserved, we examined if these candidate genes are conserved at a further evolutionary distance by identifying their orthologs in the genome of *Drosophila melanogaster*. This analysis again revealed that the overlapping mouse and human candidate genes were more often conserved throughout evolution as compared to the genes uniquely identified with a mouse or human signature (Fig 1C). Furthermore, the overlapping genes included members of a core cardiogenic transcriptional network including the TFs NKX2-5 (Tinman, Tin in *Drosophila*), GATA4 (Pannier in *Drosophila*), ISL1 (Tailup in *Drosophila*), Hand-1/-2 (Hand in *Drosophila*), TBX5 (Dorsocross genes in *Drosophila*) and TBX20 (H15 or midline in *Drosophila*) (S1 Table) [27]. Lastly, gene ontology analysis revealed that the overlapping candidate cardiogenic genes were enriched for signaling and transcriptional functions as well as cardiac developmental pathways, whereas the genes unique to the mouse or human signature were not enriched for these functions (Fig 1D and S1 Table). Interestingly, the human alone genes were enriched for Wnt signaling and cell death functions whereas the mouse alone genes were enriched for angiogenesis functions (S1 Table). This result suggests that the differentiation of mouse and human ESCs along the cardiac lineage utilizes distinct signaling pathways that may lead to unique cellular outcomes. In total, these results show that the overlapping mouse and human genes are the best candidates for having cardiac regulatory activity.

### Functional screen of mammalian candidate cardiogenic genes in *Drosophila*

The preceding analyses revealed that the overlapping genes between human and mouse are the best candidates for having cardiogenic activity, with the majority conserved in *Drosophila*. Numerous studies have recently shown the utility of functional genomic screens in *Drosophila* to inform candidate gene function in mammals [28,29]. Thus, to identify if the candidate cardiogenic genes are functional, we first screened the function of their orthologs in *Drosophila* by targeting RNAi against a group of candidate genes to the dorsal mesoderm in which the *Drosophila* heart develops (see Materials and Methods).

**Fig 2:**
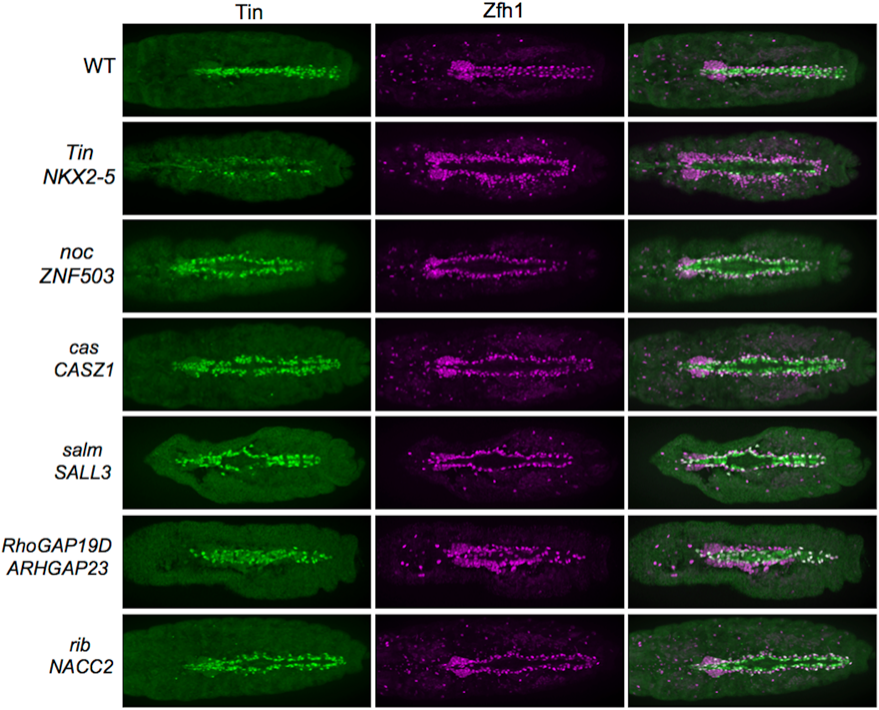
Screening orthologs of the mammalian candidate genes in *Drosophila* identifies regulators of heart development. Stage 16 *Drosophila* embryos were collected from flies expressing Hand-Gal4, TinD-Gal4, and UAS-dcr2 to flies expressing UAS-RNAi against the indicated gene (with the human ortholog also indicated) or yw (wild-type) and stained with the cardiac markers Tin (green) and Zfh1 (magenta). Genes tested were randomly selected based on public availability of siRNA stocks. Results shown are representative of at least 10 embryos. Embryo images are shown for 5 genes, with the entirety of the Drosophila screen available in S1 Table. Many siRNA stocks yielded no observable cardiac phenotypes arguing against toxicity of siRNAs creating cardiac phenotypes (S1 Table).

The heart in *Drosophila* is a linear tube composed of an inner layer of contractile cardial cells (CCs) and an outer layer of non-muscle pericardial cells (PCs) and is most similar to the mammalian heart at the linear tube stage of development (see Fig 2, WT) [30]. This correlation is not only structural but also has functional similarities since specification of cardiac progenitors by a conserved signaling and transcriptional network is utilized for CP specification during the linear tube stage of development [31]. In addition, the simple organization of the fly heart is advantageous in that it permits the rapid identification of LOF phenotypes as both the generation of inappropriate numbers of heart cells or a failure in heart tube closure creates readily detectable aberrations in heart tube organization. For example, RNAi knockdown of the NKX2-5 ortholog Tin creates abnormal numbers of PCs and CCs causing defects in heart morphology (Fig 2, Tin).

We next screened the function of the fly orthologs of 19 mammalian genes identified with both the mouse and human epigenetic gene expression signature in *Drosophila* using RNAi targeted to the dorsal mesoderm. Importantly, the reduced number of paralogs in *Drosophila* simplifies functional analyses, with these 19 mammalian genes associated with 17 fly orthologs. In total, 12 out of these 17 fly genes showed defects in the morphology of the *Drosophila* heart (Fig 2). Combining these genes with other genes characterized in the literature with cardiac phenotypes in *Drosophila* identifies 15 potential new mammalian cardiogenic genes (S1 Table) [32]. Thus, a functional screen in *Drosophila* guided by a multi-species mammalian gene expression signature can be used to identify orthologous gene function.

### ZNF503, ZEB2 and NKX2-5 knockdown in differentiating human ESCs reveals their essential role in cardiac differentiation

We next asked if these newly identified regulators of cardiogenesis are required in the human cardiac lineage. To do so, we used small molecule-mediated directed differentiation of human ESCs into cardiomyocytes (CMs) (see Materials and Methods). This protocol induces the rapid down-regulation of genes associated with pluripotency such as NANOG and POU5F1 followed by gene expression associated with the mesendodermal lineage such as MIXL1 following 2 days of differentiation (S2A Fig). At 4 days post-differentiation, gene expression associated with the cardiac lineage begins with robust activation of GATA4 followed by expression of NKX2-5, MEF2C, and TBX5 at 6 days post-differentiation (S2A Fig). This CP population leads to the production of predominantly CMs expressing myosin heavy and light chain genes and cardiac troponin (cTnT) which peak at day 10 (S2A Fig).

**Fig 3:**
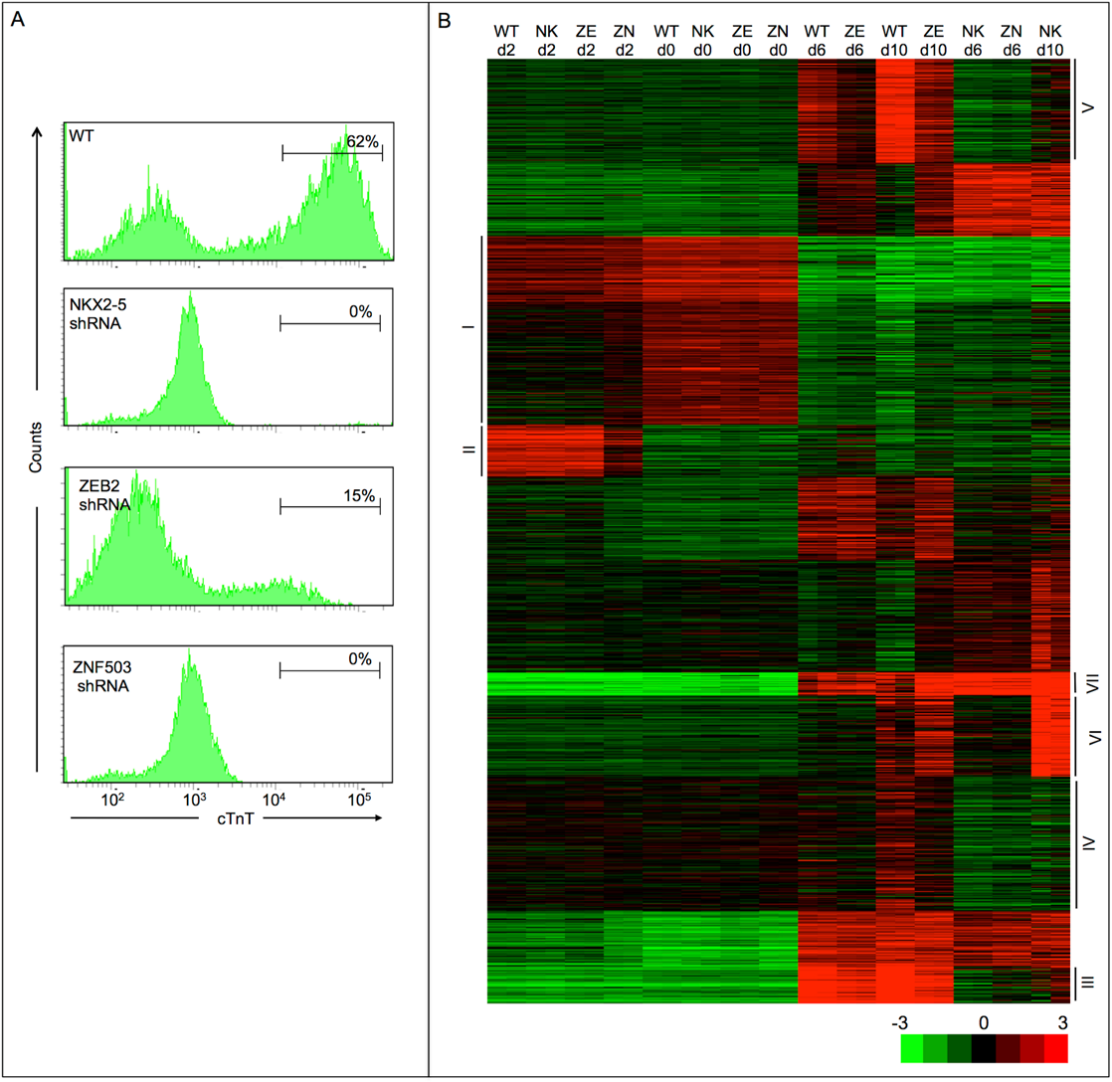
ZNF503, ZEB2 and NKX2-5 are necessary for differentiation of CMs from human ESCs. (A) Flow cytometric analysis of cTnT expression on human ESCs after 10 days of differentiation along the cardiac lineage following shRNA knockdown of the indicated genes. Representative results of five experiments. WT cells were infected with a shRNA targeting GFP or empty vector, which yielded identical results as non-infected cells. (B) Hierarchical clustering of genes showing significant gene expression changes following shRNA knockdown of the indicated genes at the indicated time points. WT = wild-type; ZE = ZEB2 shRNA; ZN = ZNF503 shRNA; NK = NKX2-5 shRNA.

To identify if the candidate genes are essential in differentiation of the human cardiac lineage, we first examined the expression of two of these genes in CM differentiation, ZNF503 and ZEB2. These genes began showing expression at day 2 which peaked 4 days after the initiation of differentiation when the cardiac lineage was first selected and this expression remained throughout differentiation (S2B Fig). To interrogate the function of ZNF503 and ZEB2 in CM differentiation, we used shRNA-mediated knockdown in differentiating human ESCs. As a positive control, we also examined the function of NKX2-5, a gene with a well-known role in heart development and CM differentiation [33]. Knockdown of these genes using shRNA reduced mRNA expression of the target genes by at least 90% compared to wild-type (WT) (S3 Fig). We used flow cytometry to monitor the effects of the knockdown of these genes following directed differentiation along the cardiac lineage. This study revealed that ESCs in which NKX2-5 was knocked down failed to generate cTnT-expressing CMs (Fig 3A). Similarly shRNA knockdown of ZNF503 did not produce cTnT-expressing CMs whereas ZEB2 knockdown reduced the percentage and expression level of cTnT-expressing CMs (Fig 3A). Further, differentiating WT ESCs produced CMs that contracted synchronously beginning at day 7–8. However, shRNA knockdown of ZNF503, NKX2-5 or ZEB2 ESCs failed to generate contractile CMs (data not shown). Similar results were seen if cultures were differentiated for longer times (14 days) suggesting that the effects of ZNF503, ZEB2 and NKX2-5 knockdown are not simply due to slower differentiation, proliferation or metabolic activity of the cells (data not shown). In addition, there was no difference in cell numbers and viability at days 0, 2 and 6 for all groups and at day 10 for ZEB2 and NKX2-5 shRNA conditions. However, ZNF503 knockdown cells did show high levels of cell death at day 10 which likely reflects the culture conditions for CMs is inappropriate for the ZNF503 knockdown cells. In total, these results show that ZNF503 and ZEB2 play an essential role in human CM differentiation from ESCs.

### Transcriptome profiling uncovers gene expression patterns that underlie human CM differentiation

To characterize the molecular mechanisms through which ZNF503, ZEB2 and NKX2-5 induce specification and differentiation of CMs, RNA-seq was performed on biological replicates at 0, 2, 6 and 10 days of differentiation in WT human ESCs and those in which ZN503, ZEB2 and NKX2-5 were depleted with shRNA. All samples yielded over 30 million raw reads with a high mapping rate aside from the day 10 samples for ZNF503 shRNA knockdown which had lower numbers of raw reads and mapping rate. This finding reflects the isolation of poor quality RNA due to the high amount of cell death at day 10 in ZNF503 shRNA cells that was detected in multiple samples. These data were subsequently discarded from further analysis. Differentially-expressed genes were identified between WT and shRNA knockdown conditions, and k-means hierarchical clustering was used to identify gene expression signatures associated with perturbation of gene function (Fig 3).

The clustering data reveals multiple gene expression clusters associated with the differentiation of human ESCs along the cardiac lineage (Fig 3B). In particular, a cluster of genes is enriched at day 0 of differentiation and becomes repressed as cells differentiate along the cardiac lineage (cluster I). We next used Reactome to identify the biological pathways, reactions and processes enriched amongst this gene set [10]. This analysis revealed an enrichment for genes involved in transcriptional regulation of pluripotent stem cells and developmental biology, which is consistent with the presence of genes associated with pluripotency, including POU5F1, NANOG, SOX2 and FOXO1 in this cluster (S2 Table). These results shows that gene expression patterns associated with pluripotency are initially repressed across all genetic perturbations.

**Fig 4:**
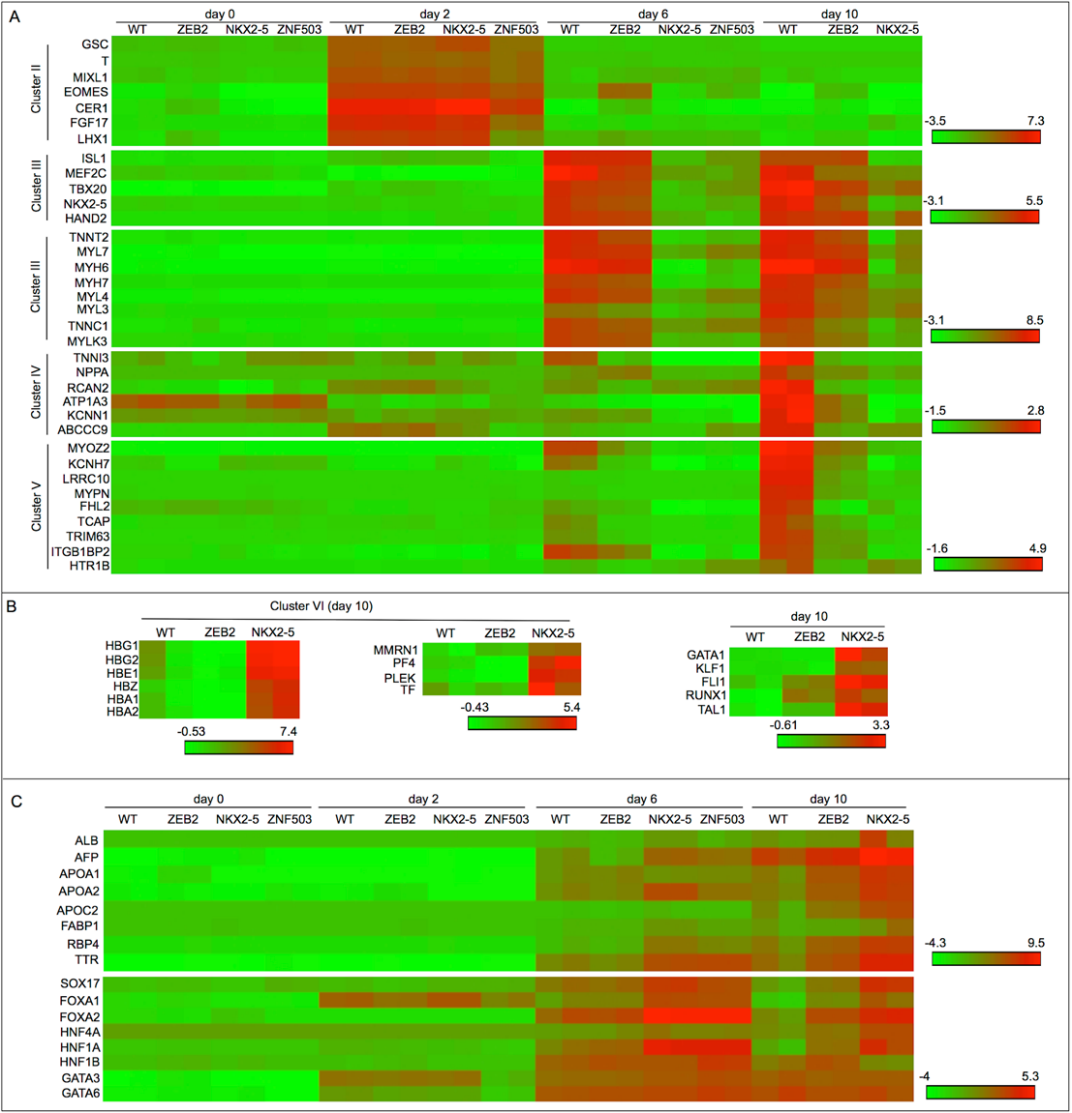
Transcriptome analysis reveals a role for cardiogenic TFs in activation of cardiac genes and repression of alternative cell fates. Matrix representations of gene expression patterns at different time points of differentiation along the cardiac lineage are shown for WT or shRNA-treated ESCs. (A) Gene expression patterns of mesendodermal (cluster II) and cardiac lineage genes (clusters III, IV, and V) at the indicated time points. (B) Gene expression patterns of the indicated haematopoietic genes at day 10. (C) Gene expression patterns of liver and definitive endodermal genes at the indicated time points.

A second cluster of genes is normally repressed at day 0 and becomes active at day 2 of differentiation, followed by a decrease that begins at day 6 (cluster II). This cluster includes genes associated with mesodermal and/or mesendodermal formation including GSC, T, MIXL1, EOMES, CER1, FGF17 and LHX1 (Fig 4A and S2 Table). In addition, biological pathway analysis revealed an enrichment for genes related to differentiation as well as FGFR and WNT signaling activity which are required for mesendodermal formation [34] (S2 Table). These results show that the initial events in the directed differentiation along the cardiac lineage is occurring normally in NKX2-5, ZNF503 and ZEB2 shRNA cells, suggesting that the defects in CM formation are due to defects in later cell fate specification events.

After 6 days of differentiation, a gene expression cluster (III) shows increased expression in WT cells but no activity in NKX2-5 and ZNF503 shRNA-treated cells. This cluster is enriched for genes involved in striated muscle contraction and mitochondrial biogenesis that are essential features of CMs (S2 Table). This cluster also includes essential cardiogenic TFs including ISL1, MEF2C, TBX20, NKX2-5 and HAND2 [35], and CM contractile proteins including myosin heavy (MYH6, MYH7) and light chain genes (MYL7, MYL4, MYL3) and troponins (TNNT2, TNNC1) (Fig 4A and S2 Table). Thus, the inability of ZNF503 and NKX2-5 shRNA cells to differentiate into CMs is correlated with an inability to activate this core set of conserved TFs that ultimately control cardiac cell fates and the expression of downstream target genes that encode contractile and structural proteins [35]. Although many of these genes are activated to similar levels in WT and ZEB2 shRNA cells, there are a few genes with attenuated expression including those involved in later differentiation events including MEF2C and a few myosin heavy and light chain genes (Fig 4B). This outcome suggests that the inability to activate terminal CM differentiation programs may underlie the failure of ZEB2 shRNA cells to differentiate into contractile CMs.

In support of this hypothesis, two additional gene expression clusters (IV and V) become active in WT cells at day 10 following cardiac differentiation but fail to upregulate in ZEB2 and NKX2-5 shRNA cells (Figs 3B and 4A). Biological pathway analysis showed an enrichment for striated muscle contraction, PKA activation and the calmodulin pathway in cluster IV and HCN channels and potassium channels in cluster V which are all essential molecular pathways in CM contractility [36,37] (S2 Table). Representative gene expression patterns for cluster IV and V genes are shown in Fig 4A. These results identify an inability of ZEB2 shRNA cells to activate terminal differentiation genes encoding essential cardiac contractile components and proteins involved in the excitatory system that characterizes CMs.

### ZNF503 and NKX2-5 may repress alternative cell fates

The above results show that NKX2-5 and ZNF503 shRNA cells fail to become CMs. To characterize the identity of the cells in these differentiated cultures, we examined their gene expression patterns. A cluster of genes (VI) are robustly increased at day 10 in NKX2-5 shRNA cells but weakly in WT cells (Fig 3B). Interestingly, biological pathway analysis reveals an enrichment for erythrocyte and platelet functions (S2 Table), suggesting that NKX2-5 shRNA cells differentiated along the haemaotpoietic lineage. In support of this conclusion, day 10 NKX2-5 shRNA cells expressed high levels of hemoglobin subunits (HBG1, HBG2, HBE1, HBZ, HBA1, HBA2) indicative of erythrocytes and components involved in platelet degranulation and hemostasis (MMRN1, PF4, PLEK, TF) (Fig 4B and S2 Table). In support of increased specification of the haematopoietic lineage of NKX2-5 shRNA cells, these cells increased expression of essential TFs involved in the specification of erythrocytes and platelets including GATA1, KLF1, FLI1, RUNX1 and TAL1 (Fig 4B) [38]. The confirmation that NKX2-5 shRNA cells increased specification along the haematopoietic lineage does require additional experimentation, including single cell analyses of cell surface markers characteristic of erythrocytes and platelets. However, a role for NKX2-5 in the repression of the haematopoietic/erythroid molecular program is consistent with a previous description in mouse development and embryonic stem cell differentiation [39].

Interestingly, biological pathway analysis of cluster VI genes revealed an enrichment for genes involved in lipid digestion, mobilization and transport genes characteristic of hepatic cells (S2 Table). In addition, a seventh cluster of genes (VII) is enriched in ZNF503 and NKX2-5 shRNA cells and these genes are similarly enriched for lipid metabolic functions (S2 Table). This suggests that NKX2-5 and ZNF503 shRNA cells are increasing expression of genes characteristic of hepatic cells. In support, we found that genes characteristic of the hepatocyte cell lineage such as ALB, APOC2 and FABP1 in cluster VI and AFP, APOA2, RBP4 and TTR in cluster VII are upregulated in NKX2-5 and ZNF503 shRNA cells (Fig 4C) [40]. To further characterize the increased expression of a hepatic cell molecular program in the absence of NKX2-5 and ZNF503, we examined the time course of expression of genes involved in endoderm formation [41]. We show robust activation of most definitive endoderm genes at day 6 of differentiation, including SOX17, FOXA1, FOXA2 and HNF1A in NKX2-5 shRNA cells that mostly diminish by day 10 of differentiation [41]. Other definitive endodermal genes including GATA3 and GATA6 are increased in all cells which likely reflects their additional activity in the cardiac lineage [42,43]. Further, FOXA2 and HNF1A, TFs expressed in differentiated hepatocytes, are highly expressed in NKX2-5 and ZNF503 shRNA cells [40,41]. In total, these results show that an important role of the cardiogenic genes NKX2-5 and ZNF503 is the repression of gene expression patterns characteristic of endodermal cell fates including hepatocytes.

## Conclusions

We show that the distribution of histone marks found within differentiating human and mouse ESCs predicts genes critical for CM differentiation, with the best predictions provided by the overlapping mouse and human candidates. A recent study used whole exome sequencing of patients with congenital heart defects (CHD) to show that ~2% of the human genome is associated with CHD [44]. We show that 36 of 98 (36.7%) overlapping candidate genes are associated with exome mutations that lead to CHD, thereby underscoring the importance of integrating orthologous data sets to improve candidate gene expression models. Furthermore, a recent study showed that conserved epigenetic signatures are enriched for genetic variants associated with Alzheimer’s disease [45]. An RNAi-based screen of the *Drosophila* orthologs of the mammalian genes uncovered multiple novel cardiogenic regulators. When two of these newly-identified cardiogenic genes were tested in differentiating human ESCs, both were shown to play essential roles in the specification of the cardiac lineage. These results confirm the value in integrating functional data sets from multiple species to identify the most conserved developmental pathways. We further show that ZEB2 activates terminal CM differentiation events required for CM structure and function whereas ZNF503 and NKX2-5 are required for specification of the cardiac lineage. As mutations in ZEB2 have been reported in Mowat-Wilson syndrome, a congenital anomaly that presents with CHD, this newly-discovered role of ZEB2 provides novel insights into its role in CHD [46]. Furthermore, this work describes an uncharacterized role for ZNF503 in specification of the cardiac lineage. In addition, we show that an important factor of cardiac lineage specification in human ESCs is the repression of alternative gene expression programs by TFs such as ZNF03 and NKX2-5. In total, these results demonstrate the utility of combining orthologous genomic data sets with empirical testing and transcriptome profiling in differentiating ESCs to identify the genes and gene regulatory networks that characterize the human cardiac lineage.

## Supporting Information

S1 Fig Histone mark distribution of potential cardiogenic genes. The distribution of the indicated histone modifications to the genomic region surrounding human ZNF503 (A) and ZEB2 (B) at the ESC or CP state. Genomic coordinates (hg19) are indicated for human chromosome 10 (A) and 2 (B). Brackets indicate scale of peak.
(TIF)

S2 Fig Gene expression across differentiation of human ESCs along the cardiac lineage. (A) Real-time PCR analysis of the indicated genes at days 0, 2, 4, 6 and 10 and for the candidate cardiogenic genes ZEB2 and ZNF503 (B) following differentiation of H1 ESCs along the cardiac lineage. Gene expression was normalized to the housekeeping gene GAPDH. The comparative Ct method was used to compare expression differences between day 0 and the other days. Error bars represent standard deviation of triplicate PCR reactions. Representative results of 2 experiments.
(TIF)

S3 Fig shRNA knockdown of NKX2-5, ZNF503 and ZEB2 in human ESCs. Real-time PCR analysis of the indicated genes following directed differentiation of H1 ESCs along the cardiac lineage in the presence of shRNAs targeting NKX2-5, ZNF503 and ZEB2. Gene expression was normalized to the housekeeping gene GAPDH. The comparative Ct method was used to compare expression differences between WT to shRNA knockdown. Representative results of 2 experiments.
(TIF)

S1 Table Candidate cardiogenic genes identified based on an epigenetic gene expression of human and mouse ESCs. Genes, function and gene ontology analysis is shown.
(XLSX)

S2 Table Gene expression clusters of human ESCs. Expression differences for different genes across time and genetic perturbation of H1 ESCs differentiating along the cardiac lineage. Reactome pathway analysis of those clusters is shown.
(XLSX)
