# Supplementary figures and images for: An Orthologous Epigenetic Gene Expression Signature Derived from Differentiating Embryonic Stem Cells Identifies Regulators of Cardiogenesis
Source: PLoS One. 2015 Oct 20;10(10):e0141066. doi: 10.1371/journal.pone.0141066 (PMC4617299; doi:10.1371/journal.pone.0141066)

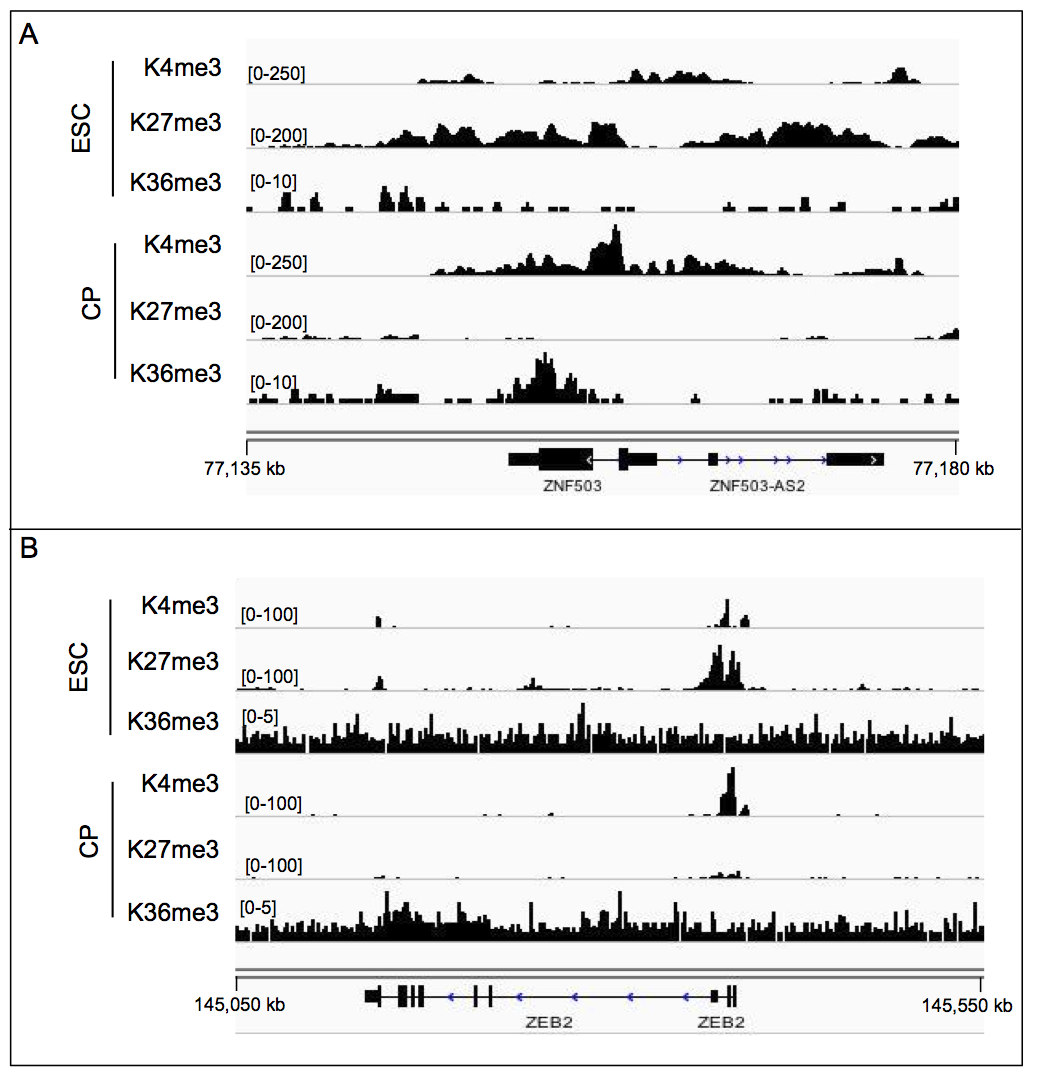

Supplement: S1 Fig — The distribution of the indicated histone modifications to the genomic region surrounding human ZNF503 (A) and ZEB2 (B) at the ESC or CP state. Genomic coordinates (hg19) are indicated for human chromosome 10 (A) and 2 (B). Brackets indicate scale of peak. (TIF) [file pone.0141066.s001.tif]

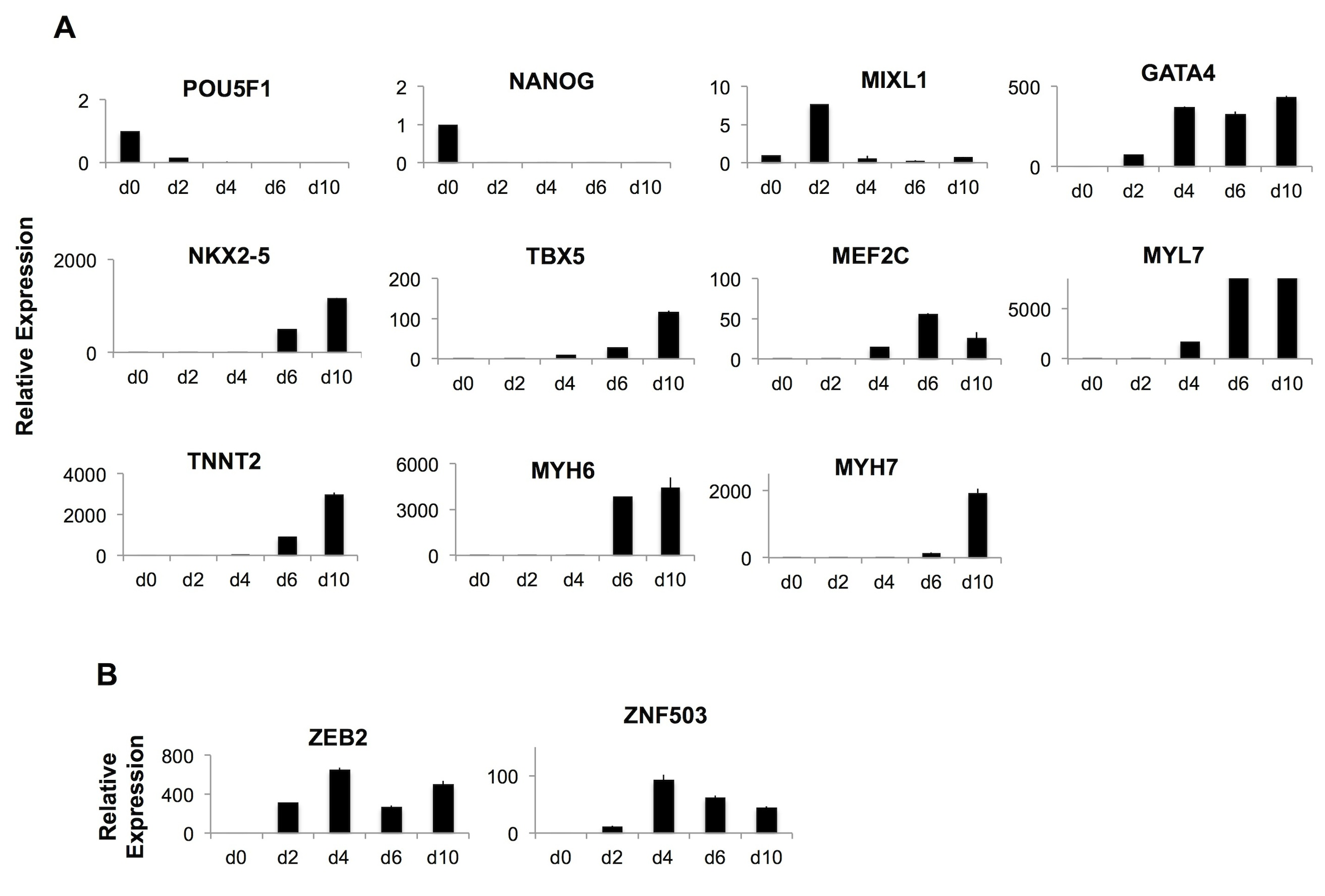

Supplement: S2 Fig — (A) Real-time PCR analysis of the indicated genes at days 0, 2, 4, 6 and 10 and for the candidate cardiogenic genes ZEB2 and ZNF503 (B) following differentiation of H1 ESCs along the cardiac lineage. Gene expression was normalized to the housekeeping gene GAPDH. The comparative Ct method was used to compare expression differences between day 0 and the other days. Error bars represent standard deviation of triplicate PCR reactions. Representative results of 2 experiments. (TIF) [file pone.0141066.s002.tif]

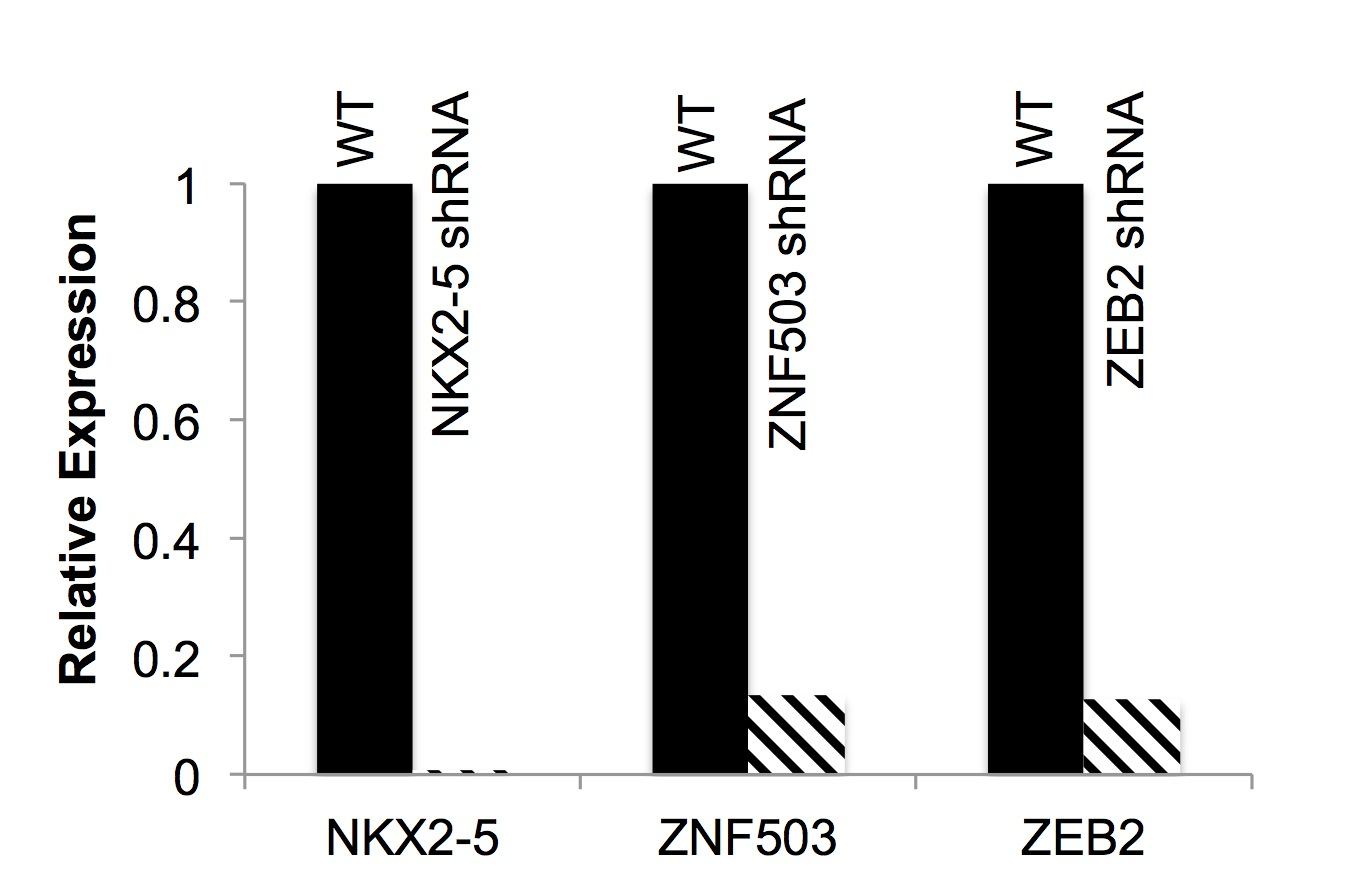

Supplement: S3 Fig — Real-time PCR analysis of the indicated genes following directed differentiation of H1 ESCs along the cardiac lineage in the presence of shRNAs targeting NKX2-5, ZNF503 and ZEB2. Gene expression was normalized to the housekeeping gene GAPDH. The comparative Ct method was used to compare expression differences between WT to shRNA knockdown. Representative results of 2 experiments. (TIF) [file pone.0141066.s003.tif]
